# Prognosis after steroid pulse therapy and seasonal effect in acquired idiopathic generalized anhidrosis

**DOI:** 10.1111/1346-8138.15666

**Published:** 2020-11-04

**Authors:** Tadatsune Iida, Michiko Nakamura, Minako Inazawa, Takichi Munetsugu, Makiko Nishida, Tomoko Fujimoto, Yoshiyuki Sasaki, Yuichiro Ohshima, Yoshihiko Nakazato, Takeshi Namiki, Hiroo Yokozeki

**Affiliations:** ^1^ Department of Dermatology Graduate School of Medical and Dental Sciences Tokyo Medical and Dental University Tokyo Japan; ^2^ Department of Maxillofacial Surgery Graduate School of Medical and Dental Sciences Tokyo Medical and Dental University Tokyo Japan; ^3^ Department of Dermatology Aichi Medical University School of Medicine Nagakute Japan; ^4^ Department of Neurology Saitama Medical University Moroyama Japan

**Keywords:** cohort studies, hypohidrosis, sweat glands, temperature, urticaria

## Abstract

Acquired idiopathic generalized anhidrosis is a rare disease with unknown etiology. Sudden loss of sweating function adversely affects young patients’ quality of life. Although systemic corticosteroid therapy is the most frequently reported treatment for the disease, its effectiveness is controversial because of the risk of recurrence. To assist clinical decision‐making regarding whether to use steroids, we investigated the treatment responsiveness and recurrence rates in patients undergoing steroid pulse therapy and explored factors affecting these rates. We retrospectively collected data of 124 patients who received steroid pulse therapy to calculate the rate of responsiveness to the therapy. We also conducted a time‐to‐event analysis in a cohort of 57 patients who responded to steroid pulse therapy to estimate the recurrence rate after the therapy. As a result, the response and recurrence rates were 73% and 48%, respectively. Recurrence occurred within 1 year in most patients. The overall effectiveness of steroid pulse therapy was estimated to be 57% considering the recurrence rate. A delay from onset to treatment and younger age appeared to be negative factors for effectiveness. Moreover, we found a significant seasonal effect on both treatment and recurrence: autumn was the worst season for acquired idiopathic generalized anhidrosis in Japan. Our study revealed that steroid pulse therapy can be expected to be effective in half of treated patients. We recommend starting the therapy promptly after the diagnosis; however, it is also worth considering the season for treatment planning.

## INTRODUCTION

Acquired idiopathic generalized anhidrosis (AIGA) is a rare and refractory disease with unknown etiology and mainly occurs in young men.^1^ The main symptom is impairment of sweating, which limits patients’ activity in hot environments and strongly affects quality of life.^2^ Most cases of AIGA have been reported in Asia, especially Japan.^1^, ^3^, ^4^ The cause of the disease has not been determined, but studies have suggested that immunological mechanisms may be involved in the impairment of sweating.^5^, ^6^, ^7^ Although some cases of AIGA are cured spontaneously, most appear to become intractable after a certain time without treatment.^3^, ^8^, ^9^

Systemic steroid therapy is the most frequently reported treatment for AIGA, but its effectiveness has not been sufficiently evaluated. Several reports have indicated considerable effectiveness of steroids for improvement of sweating function,^5^, ^6^, ^8^, ^10^ but complete ineffectiveness has also been reported in some patients.^3^, ^11^, ^12^ Furthermore, a high recurrence rate after the therapy has been reported.^8^, ^13^ All such information to date has been obtained from case reports or small case series;^8^, ^14^, ^15^ no study has been performed to estimate treatment effectiveness considering both the response and recurrence rates. In addition, although several factors have been suggested to influence the prognosis, including age, sex and duration from onset to treatment, these findings were not supported by statistics.^3^, ^13^ Knowledge of the precise treatment effectiveness with consideration of the recurrence rate as well as identification of prognostic factors would be helpful for determining whether to use steroids in patients with AIGA.

To estimate the true effectiveness of steroid therapy in patients with AIGA, we collected data from 124 patients treated with steroid pulse therapy and retrospectively examined their responsiveness to the treatment. We then investigated the recurrence rate among 57 patients who responded to the therapy. Further, we explored prognostic factors affecting the treatment effectiveness.

## METHODS

### Ethics

We conducted this study according to the principles of the Declaration of Helsinki. The study was approved by the ethics committees of Tokyo Medical and Dental University, Saitama Medical University, and Aichi Medical University hospitals.

### Responsiveness to steroid pulse therapy

To assess the responsiveness to the therapy, we retrospectively collected data from all patients with AIGA who received pulse therapy with 3‐day i.v. methylprednisolone (0.5–1.0 g/day) at Tokyo Medical and Dental University hospital from April 2008 to May 2019, Saitama Medical University hospital from January 2015 to December 2018, and Aichi Medical University hospital from November 2015 to March 2017. The patients’ age at start of treatment, sex, medical history, diagnostic and follow‐up examinations, and clinical course were obtained from the medical records. We confirmed the diagnosis of AIGA in accordance with the Japanese guideline.^1^ Patients were judged as responders to the therapy if the body surface area with perspiration had increased by more than 25% at 1 month after the pulse therapy. This area of increased perspiration was estimated based on a medical interview 1 month after the therapy and, in some patients, a subsequent starch‐iodine test (the accuracy of interviews is assessed in Table S6). When patients received multiple courses of pulse therapy, those who met the criteria at least once were grouped as responders; the remaining patients were grouped as non‐responders. We adopted 25% as the threshold according to the criteria for scoring disease severity described in the Japanese guideline (area of hypohidrosis or anhidrosis scored as follows: 1, 25%–49%; 2, 50%–74%; and 3, 75%–100%).^1^ This score reportedly has a good correlation with quality of life.^2^ In this analysis, we did not consider whether the patients developed recurrence after the therapy; that is, we grouped all of the patients with recurrence as responders. We also explored whether the patients’ characteristics affected the responsiveness.

**Figure 1:**
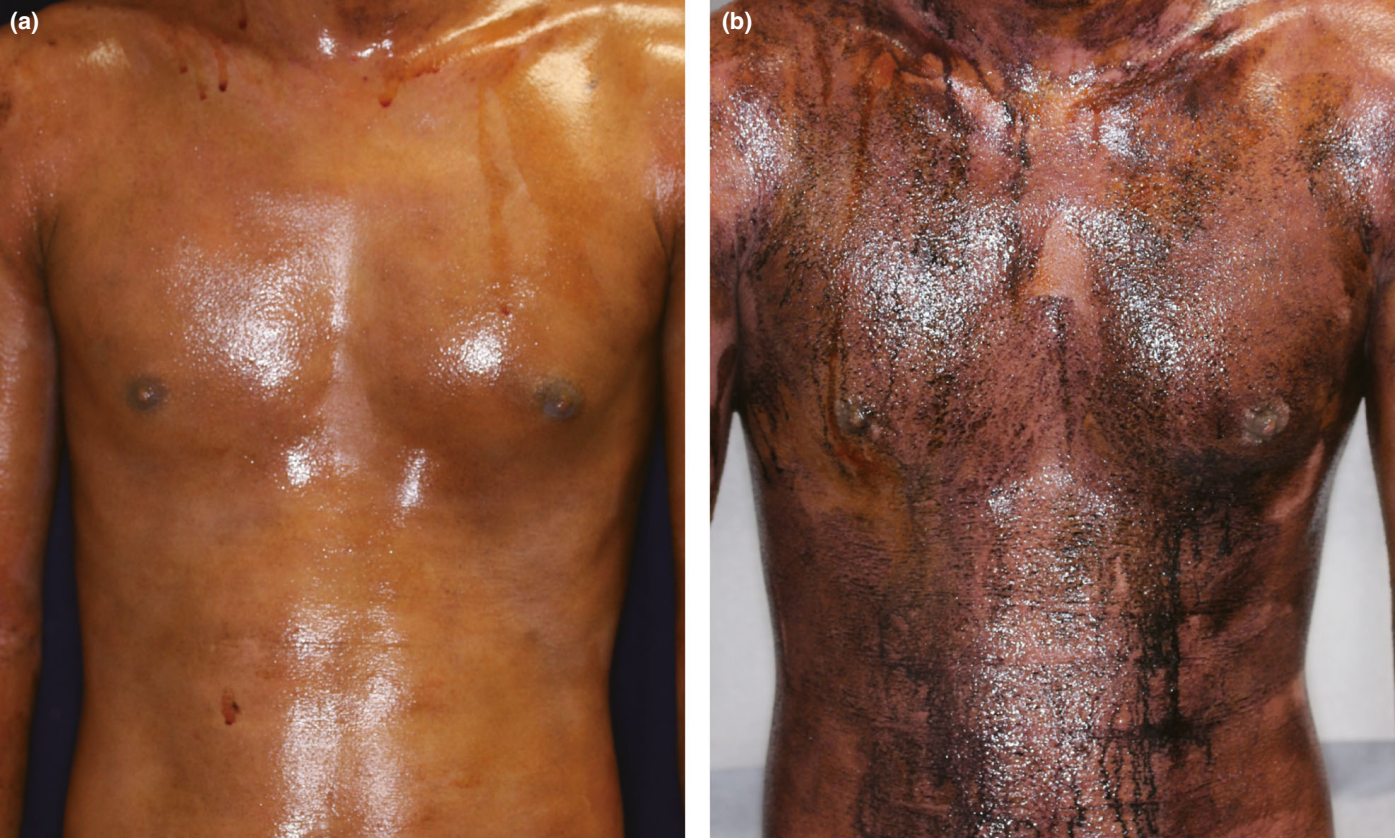
Representative images of a patient who responded to steroid pulse therapy. (a) Image of Minor’s test (starch‐iodine test) of a patient with acquired idiopathic generalized anhidrosis before treatment. There was no obvious change in color after 15 min in a sauna at 65°C, indicating reduction of sweating function. (b) Image of Minor’s test of the same patient at 1 month after steroid pulse therapy. The color changed to black after 15 min in a sauna at 65°C, indicating recovery of sweating function.

We examined the effect of each course of pulse therapy by using the data of patients from Tokyo Medical and Dental University hospital. We analyzed the influence of several factors on the responsiveness to each course of pulse therapy, including post‐treatment (oral prednisolone after the 3‐day pulse), number of courses, season and interval from the previous course (Table 2). We compared the rate of responsiveness between every possible pair of months for more precise investigation on the seasonal effect (Fig. 1b, Table S2). We also computed the correlation between the monthly rate of effectiveness and the mean temperature, and the correlation between the monthly rate of effectiveness and the mean humidity in Tokyo, Japan, in 2019.^16^ The rate of responsiveness for each number of courses of pulse therapy was calculated separately for responders without recurrence (further grouped according to the total number of courses performed), responders with recurrence and non‐responders (Table S3). We also investigated adverse effects for each course of steroid pulse therapy (Table S4).

### Recurrence after steroid pulse therapy

To estimate the recurrence rate after successful steroid pulse therapy, we conducted a retrospective cohort study using the data of the patients who had responded to the pulse therapy from Tokyo Medical and Dental University hospital. Time zero was set to the 3rd (final) day of the last course of steroid pulse therapy, and the observation was censored at the time of recurrence or the end of the observation period. Patients were judged to have developed recurrence if the area with perspiration had decreased by more than 25% since the state of remission according the criteria for scoring disease severity.^1^ When a patient also received pulse therapy after recurrence, time zero was set to the last day of pulse therapy before the recurrence, and only the initial recurrence was analyzed. The recurrence rate was estimated by the Kaplan–Meier method. We also explored whether the patients’ characteristics affected the recurrence rate by univariate analyses (log–rank test). The optimal threshold value of age was determined by receiver–operator curve analysis with recurrence as the negative label (Fig. S1). A Cox proportional hazards model was applied to compare the young and older groups. We confirmed the assumption of proportional hazards by the log‐minus‐log of transformed Kaplan–Meier estimates of the survival function (Fig. S2). We compared the patient characteristics between the young and older groups to identify the potential cofounders (Table S5).

The recurrence rate for each season was estimated based on the data from Tokyo Medical and Dental University hospital. According to the Japanese climate, we defined spring, summer, autumn and winter as March–May, June–August, September–November and December–February, respectively. The recurrence rate for each season was calculated as:

(1) $$\text{Rate =}\;\frac{N_{1}}{N_{1}+N_{2}},$$

where *N*_1_ denotes the number of patients who developed recurrence during the season and *N*_2_ denotes the number of patients who completed observation without recurrence during the season. We deleted duplicate counts of seasons for patients with more than 1 year of follow up.

### Estimation of overall effectiveness

We estimated the overall rate of effectiveness of steroid pulse therapy with the following equation:

(2) $$\text{Effectiveness}=p_{1}\times \left(1‐p_{2}\right)+p_{1}\times p_{2}\times p_{3},$$

(3) $$p_{3}=\frac{n_{1}}{n_{2}},$$

where *p*_1_ denotes the initial response rate for the steroid pulse therapy (Table 1), *p*_2_ denotes the estimated recurrence rate during 12 months after the therapy (Figure 3a), *n*_1_ denotes the number of patients who responded to the pulse therapy performed after the first recurrence with no subsequent recurrence, and *n*_2_ denotes the total number of patients who received pulse therapy after recurrence.

We also estimated the overall rate of effectiveness by a retrospective cohort study using the data of all patients who had undergone pulse therapy at Tokyo Medical and Dental University hospital (Fig. S4). Time zero was set as the starting day of the first course of steroid pulse therapy. The observation period ended on the day of the last course of steroid pulse therapy for “cured” patients, whose remission periods were confirmed to be longer than 11 months. For “not cured” patients, including responders with a confirmed remission period that did not reach 11 months and non‐responders, the end of the observation period was set at 11 months before the last consultation day. We uniformly set the end at 11 months before the last consultation day to exclude the uncertain period during which we could not judge whether the patients had been cured and to minimize selection bias. We excluded data from patients whose observation period was calculated as less than 0 months after the subtraction of 11 months. The cure rate was estimated by the Kaplan–Meier method with not cured as a survival function.

### Statistical analysis

**Figure 2:**
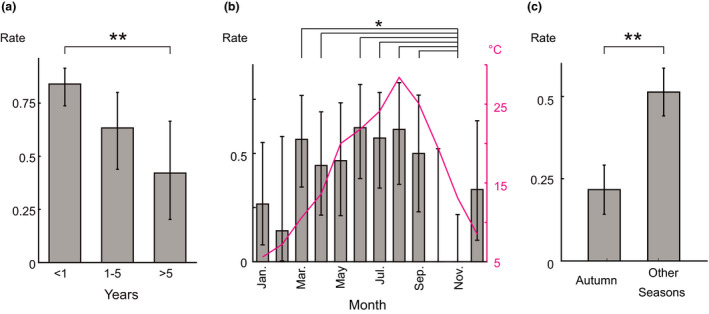
Factors influencing responsiveness to steroid pulse therapy. (a) Rate of response to steroid pulse therapy with patients grouped according to the delay from onset to start of therapy. Patients with a shorter delay showed a better response. Error bar indicates 95% confidence interval (CI). Number of patients: 75 (within 1 year), 30 (1–5 years) and 19 (>5 years). **P = 0.001, Fisher’s test with Benjamini–Hochberg adjustment (overall Fisher’s test, *P* < 0.001). (b) Monthly rate of response to an individual course of steroid pulse therapy. The plot in magenta indicates the mean temperature of each month in Tokyo, Japan, in 2019. The rate in November (late autumn in Japan) was lower than that in spring and summer. Error bar indicates 95% CI. Number of courses of pulse therapy: 19 (January), 8 (February), 36 (March), 26 (April), 22 (May), 34 (June), 33 (July), 29 (August), 21 (September), 5 (October), 15 (November) and 16 (December). *P < 0.05, Fisher’s test with Benjamini–Hochberg adjustment (overall Fisher’s test, *P* < 0.001; the *P*‐values of all pairs of months are listed in Table S2). (c) Rate of response to an individual course of steroid pulse therapy in patients who received the therapy in both autumn and the other seasons. Autumn refers to September–November. Bar indicates mean rate of response and error bar indicates standard error. Number of patients: 30. **P = 0.004, paired *t*‐test.

**Figure 3:**
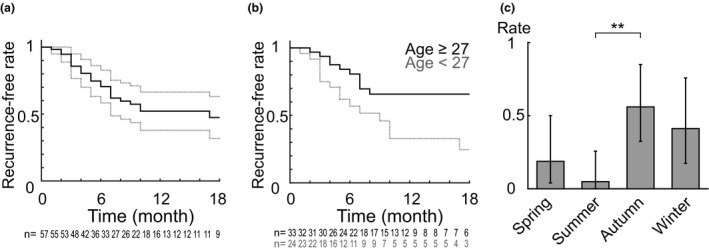
Recurrence rate after steroid pulse therapy and influential factors. (a) Kaplan–Meier plot of recurrence‐free time after steroid pulse therapy (continuous plot). The rate reached approximately 50% at month 11 and almost plateaued thereafter. Dotted plots indicate 95% confidence interval (CI). Numbers of patients at each time are indicated in the lower part. (b) Kaplan–Meier plots of recurrence‐free time in young (aged <27 years, magenta) and older (aged ≥27 years, cyan) groups of patients. The young group showed a higher rate of recurrence (*P* = 0.015, log–rank test). Numbers of patients at each time are indicated in the lower part. (c) Recurrence rates in each season. The risk of recurrence after steroid therapy in autumn was higher than that in summer. Spring, March–May; summer, June–August; autumn, September–November; winter, December–February. Error bar indicates 95% CI. Number of patients: 32, 41, 50 and 34, respectively. **P = 0.007, Fisher’s test with Benjamini–Hochberg adjustment (overall Fisher’s test, *P* = 0.003).

We performed statistical analyses by Fisher’s test (Tables 1,2, Table S4, Fig. 2a), the Mann–Whitney *U*‐test (Tables 1,2, Table S4), paired *t*‐test (Fig. 2c), the log–rank test (Fig. 3b) and the methods specified in the main text. The Benjamini–Hochberg procedure^17^ was utilized with Fisher’s test for multiple hypothesis testing (Figs 2ab,3c, Table S2). For data processing including computing *P*‐values and visualization, we used MATLAB version 2019a software (MathWorks, Natick, MA, USA); for multiple hypothesis testing, however, we used R version 4.0.0 software^18^ with the RVAideMemoire package^19^.

## RESULTS

### Responsiveness to steroid pulse therapy

For precise evaluation of the effectiveness of systemic steroid therapy for AIGA, both the response rate and recurrence rate must be considered. We first examined the response rate to steroid pulse therapy for AIGA regardless of recurrence. We retrospectively collected data of 124 patients with AIGA who received steroid pulse therapy at three academic medical centers (Table S1). The male : female ratio of the patients was 3.6:1.0, and the median age was 32 years (interquartile range [IQR], 21–41). The overall age and sex distribution were similar to those in previous studies,^1^, ^3^, ^4^, ^8^, ^9^ but the female patients tended to be younger than the male patients (24 [IQR, 18–33] vs 33 years [IQR, 21–41]; Mann–Whitney *U*‐test, *P* = 0.033). Of the 124 patients, 90 (73%) responded to the steroid pulse therapy, recovering their perspiration function within 1 month after the therapy (Table 1, Fig. 1). This response rate to steroid pulse therapy was also in accordance with a previous review of case reports of systemic steroid therapy for AIGA (78%, 25/32 patients).^8^ We also explored whether the patients’ characteristics affected the responsiveness and found that patients who received steroid pulse therapy earlier had a better response (Table 1, Fig. 2a).

**Table 1:** Comparison of patient characteristics between responders and non‐responders to steroid pulse therapy

	Responder	Non‐responder	Rate,^†^ %	*P* ^‡^
All	90	34	73 [64–80]	NA
Age, years	33 (19–41)	27 (21–41)	NA	0.326
Sex				
Male	72	25	74 [64–83]	0.469
Female	18	9	67 [46–83]	
Disease score^§^				
1	5	3	63 [24–91]	0.532
2	14	3	82 [57–96]	
3	71	28	71 [61–80]	
Cholinergic urticaria				
Yes	65	23	74 [63–83]	0.617
No	25	11	69 [52–84]	
Delay between onset and treatment, months	7 (4–18)	36 (3–108)	NA	**0.011**

Data are presented as *n*, median (interquartile range) or percentage [95% confidence interval].

^†^ Rate of response in the specified group. ^‡^For continuous variables, computed by Mann–Whitney *U*‐test; for categorical variables, computed by Fisher’s exact test. ^§^Scored according to body surface area of hypohidrosis or anhidrosis (1, 25–49%; 2, 50%–74%; 3, 75%–100%).^1^ *P* < 0.05 is indicated by bold. NA, not applicable.

For further exploration of factors that might affect the treatment responsiveness, we examined the effect of each course of pulse therapy because the patients often received multiple courses of pulse therapy until remission. We analyzed 184 courses of pulse therapy among the 85 patients from Tokyo Medical and Dental University hospital (average, 2.0 courses/patient; standard deviation, 1.9). The response rate to each course of pulse therapy was 43%, and the response was significantly affected by the season of treatment (Table 2). The response rate to pulse therapy in November, which is late autumn in Japan, was lower than that in spring and summer (Fig. 2b, Table S2). Figure 2(b) also shows the mean temperatures for each month in Tokyo. The response rates roughly correlated with the mean temperatures (Pearson’s correlation coefficient, *r* = 0.50; 95% confidence interval [CI], −0.1 to 0.8; *P* = 0.100). However, the response rates in spring were higher than those in autumn, although the temperatures are almost the same (discussed below). The correlation between the response rate and humidity, which is another factor that affects sweating function, was relatively weak (Pearson’s correlation coefficient, *r* = 0.30; 95% CI, −0.3 to 0.7; *P* = 0.350) (Fig. S3). We also confirmed that the response rate in autumn was lower than that in the other seasons by comparing the rates in patients who received multiple courses of pulse therapy and received the therapy in both autumn and other seasons (Fig. 2c).

**Table 2:** Responses to individual courses of steroid pulse therapy

	Effective	Ineffective	Rate,^†^ %	*P* ^‡^
All	80	104	43 [36–51]	NA
Post‐treatment^§^				
Yes	17	12	59 [39–76]	0.073
No	63	92	41 [33–49]	
No. of courses				
1st	31	47	40 [29–51]	**0.049**
2nd	28	21	57 [42–71]	
3rd	8	22	27 [12–46]	
≥4th	13	14	48 [29–68]	
Season of treatment^¶^				
Spring	28	28	50 [36–64]	**<0.001**
Summer	36	24	60 [28–72]	
Autumn	7	27	21 [9–38]	
Winter	9	25	26 [13–44]	
Interval, months⁂	2.3 (1.3–3.9)	3.2 (1.4–5.8)	NA	0.425

Data are presented as *n*, median (interquartile range) or percentage [95% confidence interval].

^†^ Rate of response in the specified group. ^‡^For continuous variables, computed by Mann–Whitney U‐test; for categorical variables, computed by Fisher’s exact test. ^§^Patients received oral steroid treatment after 3‐day pulse therapy of i.v. steroids, typically starting with 0.5 mg/kg per day of prednisolone for 2–4 weeks. ^¶^Spring, March–May; summer, June–August; autumn, September–November; winter, December–February. ⁂Interval from previous course of pulse therapy, calculated from the data of the second or later courses (number of courses of pulse therapy: 46 and 55). *P* < 0.05 is indicated by bold. NA, not applicable.

The response rate to the third course of pulse therapy was lower than that to the first and second courses, but this does not necessarily mean that the effect increased or decreased depending on the number of courses (Tables 2,S3). The response rate to the third course was lower than that to the first and second courses because a substantial number of patients with a good response had completed their therapy in the first or second course (36/57 patients, 63%). The response rate after the fourth course of pulse therapy was relatively higher than that after the third course because we stopped the therapy in most of the patients who had shown no response to the first three courses (11/14 patients, 79%). We also confirmed that there was no bias between the treatment season and the number of courses (Fisher’s test, *P* = 0.99).

Adverse effects of steroid pulse therapy were seen in 37 of the 184 courses of pulse therapy (20%). All adverse effects were mild except in one patient, who gained more than 20% weight during prolonged treatment with an oral steroid after pulse therapy (Table S4).^20^ Psychiatric disorders (especially insomnia) and gastrointestinal disorders were the most frequent adverse effects.

### Recurrence after steroid pulse therapy

To assess the true effectiveness of steroid pulse therapy, we next estimated the recurrence rate after the therapy. We conducted a time‐to‐event analysis involving all 57 patients who responded to the pulse therapy at Tokyo Medical and Dental University (Table S5). The traces were started on the last day of steroid pulse therapy and ended at recurrence (25/57, 44%) or were censored if the primary doctor decided to end follow up (5/57, 9%), the patient did not attend the return visit (12/57, 21%) or the end of the study was reached (21 May 2020) (16/57, 28%). Follow up to recurrence or at least 12 months was 74% complete (42/57) and median follow up in patients with no recurrence was 11.5 months (IQR, 6.6–19.0). Figure 3(a) shows the results of the analysis with a Kaplan–Meier plot. The estimated overall recurrence rate reached 48% at month 11 and almost plateaued at that time point.

Next, we explored whether the patients’ characteristics or other factors affected the recurrence rate. We found that younger patients had a greater tendency to relapse based on the univariate analyses (Table 3, Fig. 3b). Because the presence of cholinergic urticaria may be a confounding factor (Table S5), we used a Cox proportional hazard model with age and the presence of urticaria as independent variables to estimate the relative risk. As a result, the estimated hazard ratio was 2.5 for patients aged less than 27 years (95% CI, 1.1–5.8; *P* = 0.034) and 1.1 for patients with cholinergic urticaria (95% CI, 0.4–2.9; *P* = 0.841). Moreover, the recurrence rate was highest in autumn, which indicated that autumn is the worst season for maintaining remission as well as for achieving a good treatment effect (Fig. 3c).

**Table 3:** Estimation of recurrence rate after steroid pulse therapy and exploration of influential factors

	*n*	Recurrence rate at 12 months, %	*P* ^†^
All	57	48 [34–62]	NA
Age, years			
<27	24	67 [46–89]	**0.015**
≥27	33	34 [17–52]	
Sex			
Male	43	42 [26–59]	0.252
Female	14	64 [36–91]	
Disease score^‡^			
1 or 2	13	41 [13–69]	0.521
3	44	50 [33–66]	
Cholinergic urticaria			
Yes	39	52 [34–69]	0.374
No	18	38 [14–63]	
Delay between onset and treatment			
<12 months	38	46 [28–63]	0.777
≥12 months	19	53 [28–77]	

Data are presented as *n* or percentage [95% confidence interval].

^†^ Log–rank test.

^‡^ Scored according to body surface area of hypohidrosis or anhidrosis (1, 25%–49%; 2, 50%–74%; 3, 75%–100%)^1^. *P* < 0.05 is indicated by bold. NA, not applicable.

Finally, we estimated the net effectiveness of steroid pulse therapy considering both the response rate and recurrence rate. If we assume that a patient whose remission period is longer than 11 months is cured according to the result shown in Figure 3(a), only 21% of the patients (18/85 patients from Tokyo Medical and Dental University hospital) met the requirement. This rate is too low for estimation of the effectiveness because a certain number of patients responded to the therapy but were censored before 11 months of follow up after the therapy. We therefore estimated the effectiveness of the therapy using an indirect method; that is, by multiplying the estimated response rate and non‐recurrence rate (73% and 52%, respectively; the first term of Equation 2 in the Methods section). For better estimation, we added information about the effect of steroid pulse therapy performed after recurrence. We performed steroid pulse therapy after recurrence in 11 patients. Of those 11 patients, six (55%) recovered without subsequent relapse (median follow‐up time after the second remission, 7 months [IQR, 6–8]), three (27%) temporally responded but relapsed again, and two (18%) did not respond to the therapy anymore. Based on these data, we estimated the effectiveness of steroid pulse therapy for AIGA to be approximately 57% (the second term of Equation 2 approximates the cure rate of patients who developed recurrence). We also confirmed the estimation using the Kaplan–Meier method with adaptation to our definition of cure (55% cure rate at month 23) (Fig. S4).

## DISCUSSION

To investigate the true effectiveness of steroid pulse therapy for AIGA, we examined both the responsiveness and recurrence rate after steroid pulse therapy. The initial response rate to the therapy was 73% in our 124 patients. The estimated recurrence rate reached 48% at approximately 1 year and then plateaued based on our cohort study of 57 patients. The overall effectiveness of steroid pulse therapy was estimated to be approximately 57%. We also identified some negative factors that appear to influence the treatment effectiveness, including a longer delay between onset and treatment, and a younger age. Moreover, we revealed a strong seasonal effect on both treatment and recurrence. Our data indicate that although more than half of the patients benefited from steroid pulse therapy, there is room for improvement. Early recognition of the disease and rapid start of the therapy after diagnosis are crucial to improve patient outcomes. In addition, treatment planning should include consideration of the most effective seasons (spring and summer). When remission is achieved, intense follow up is recommended, especially from autumn to winter of the first year.

We recommend that basically all patients with AIGA should be considered for steroid pulse therapy. Systemic steroid therapy is currently the most frequently reported treatment for AIGA, but it is not always the first choice.^4^, ^9^, ^21^, ^22^ The main reasons to avoid using steroids may be the risk of recurrence, adverse effects associated with long‐term use of steroids and expectation of spontaneous remission. Indeed, one study showed that 37% of patients (5/14) developed recurrence after systemic steroid therapy.^13^ Our time‐to‐event analysis revealed a recurrence rate of 48% at 1 year. Although this rate is certainly high, our data also indicated that approximately 57% of patients appeared to be cured by 1–6 courses of steroid pulse therapy (median of two courses based on the 18 cured patients).

Considering the adverse effects of steroids, we must undoubtedly avoid inappropriate long‐term use of systemic steroids.^23^ In the present study, however, we observed recovery in many patients who had only received 3‐day pulse therapy of i.v. steroids; that is, their pulse therapy was not followed by prolonged oral steroids. No serious, irreversible adverse effects occurred in the patients who received steroid pulse therapy without subsequent therapy. Therefore, we suggest considering steroid pulse therapy without subsequent therapy at the beginning of the treatment, especially in patients with a high risk of adverse effects. However, prolonged oral steroid therapy at an adequate amount and for an adequate period of time, such as 0.5 mg/kg per day of prednisolone for less than 4 weeks, is worthy of consideration for patients with refractory disease because it seemed to be more effective than steroid pulse therapy alone in the present study (Table 2).

An expectation of spontaneous remission may also affect the decision to use steroids. Indeed, Cao and Tey^9^ reported that spontaneous remission was observed in five of 13 (38.5%) patients during several years of follow up. We might have waited for spontaneous remission if our patients had only mild symptoms; however, most of our patients had severe symptoms that restricted their activities: 80% of the patients showed severe hypohidrosis/anhidrosis (>75% of body surface area) and 71% had pain (Table 1).^2^ Steroid pulse therapy has the potential to relieve such symptoms within a few days.^8^, ^15^ The decision to delay therapy must be made only after careful consideration, and the risk of a decreased therapeutic effect must be considered.

Our exploratory analysis showed that autumn is the worst season for both the treatment outcome and recurrence. The mechanism of heat adaptation may account for this seasonal difference. Because autumn is not the coldest season, the ambient temperature should not be the only reason for the seasonal difference. Body temperature regulation by sweating reportedly differs between spring and autumn, although the ambient temperatures are almost the same in these two seasons.^24^ This fact indicates that seasonal adaptation over the course of winter and summer persists in spring and autumn. In addition, long exposure to heat, such as during summer, increases sweating function but later conversely reduces sweating function.^25^, ^26^ Failure to adapt to the seasonal temperature after a hot summer, such as an overshoot of the reduction mechanism, may account for the higher risks of ineffective treatment and recurrence in autumn.

A limitation of this study may be the high proportion of censored patients who did not attend the return visit in the time‐to‐event analysis. We expect that most of them had no recurrence because no other medical institution in our geographic location specialized in this rare disease. If most of the censored patients had no recurrence, bias might have affected the estimation of the recurrence rate and overall effectiveness of the therapy. We therefore conducted a simulation assuming that all of the patients who dropped out of the follow up had no recurrence until the end of the observation period (May 2020). The estimated recurrence rate in this simulation was 43% at 1 year (95% CI, 30–56; representing an ~5% decrease from the original estimation), and the overall effectiveness of steroid pulse therapy was 58% (representing a 1% increase). We do not believe that these biases are large enough to reduce the credibility of our study.

Another limitation of this study may be the accuracy of therapy evaluation because we did not always perform Minor’s test after steroid pulse therapy. In most cases, we conducted the primary evaluation of the therapeutic effect based on self‐assessment during outpatient care and confirmed the result by performing Minor’s test before starting the next course of pulse therapy at the time of rehospitalization. We believe that the self‐assessment of sweating function during follow up was reasonably accurate (93% accuracy compared with Minor’s test for follow up) (Table S6). However, a simpler method than Minor’s test should be used to evaluate whole‐body sweating function during the routine care of patients with AIGA.

In conclusion, our study revealed the precise effectiveness of steroid pulse therapy for AIGA considering both the response rate and recurrence rate. A longer period from onset to treatment and a younger age appear to be risk factors for poorer outcomes, but focusing on treatment in the proper season may increase the therapeutic effect. For example, we might encounter the need to start treatment for a patient with AIGA in autumn. In such cases, we should not abandon steroid pulse therapy before the arrival of spring even if the therapy shows no effect. It seems to take a couple of years to lose the responsiveness to steroid therapy (Fig. 2a). Therefore, to decrease the risk of adverse effects of steroids, it may be acceptable to reduce the frequency of pulse therapy during autumn and winter. However, we instead recommend increasing the frequency of pulse therapy during spring and summer.

## Conflict of Interest

None declared.

REFERENCES1

Munetsugu
T
, 
Fujimoto
T
, 
Oshima
Y

*et al*. Revised guideline for the diagnosis and treatment of acquired idiopathic generalized anhidrosis in Japan. J Dermatol
2017; 44: 394–400.2777463310.1111/1346-8138.136492

Munetsugu
T
, 
Fujimoto
T
, 
Satoh
T

*et al*. Evaluation of the correlation between severity of acquired idiopathic generalized anhidrosis and quality of life scores. J Dermatol
2017; 44: 747–752.2832808810.1111/1346-8138.137853

Murakami
K
, 
Sobue
G
, 
Terao
S
, 
Mitsuma
T
. Acquired idiopathic generalized anhidrosis: a distinctive clinical syndrome. J Neurol
1988; 235: 428–431.306546610.1007/BF003144884

Tay
LK
, 
Chong
WS
. Acquired idiopathic anhidrosis: A diagnosis often missed. J Am Acad Dermatol
2014; 71: 499–506.2485647810.1016/j.jaad.2014.03.0415

Ando
Y
, 
Fujii
S
, 
Sakashita
N

*et al*. Acquired idiopathic generalized anhidrosis: clinical manifestations and histochemical studies. J Neurol Sci
1995; 132: 80–83.852303610.1016/0022-510x(95)00125-l6

Fukunaga
A
, 
Horikawa
T
, 
Sato
M
, 
Nishigori
C
. Acquired idiopathic generalized anhidrosis: possible pathogenic role of mast cells. Br J Dermatol
2009; 160: 1337–1340.1941626110.1111/j.1365-2133.2009.09113.x7

Sawada
Y
, 
Nakamura
M
, 
Bito
T

*et al*. Decreased expression of acetylcholine esterase in cholinergic urticaria with hypohidrosis or anhidrosis. J Invest Dermatol
2014; 134: 276–279.2374823510.1038/jid.2013.2448

Nakazato
Y
, 
Tamura
N
, 
Ohkuma
A

*et al*. Idiopathic pure sudomotor failure: Anhidrosis due to deficits in cholinergic transmission. Neurology
2004; 63: 1476–1480.1550516810.1212/01.wnl.0000142036.54112.579

Cao
R
, 
Tey
HL
. Prognosis of acquired idiopathic generalized anhidrosis. J Ger Soc Dermatol
2017; 15: 940–945.10.1111/ddg.132962877199010

Yoritaka
A
, 
Hishima
T
, 
Akagi
K
, 
Kishida
S
. Successful steroid treatment of acquired idiopathic partial hypohidrosis. J Dermatol
2006; 33: 265–267.1667479110.1111/j.1346-8138.2006.00064.x11

Miyazoe
S
, 
Matsuo
H
, 
Ohnishi
A

*et al*. Acquired idiopathic generalized anhidrosis with isolated sudomotor neuropathy. Ann Neurol
1998; 44: 378–381.974960510.1002/ana.41044031412

Ogino
J
, 
Saga
K
, 
Kagaya
M

*et al*. Idiopathic acquired generalized anhidrosis due to occlusion of proximal coiled ducts. Br J Dermatol
2004; 150: 589–593.1503034810.1111/j.1365-2133.2004.05872.x13

Ohshima
Y
, 
Yanagishita
T
, 
Ito
K

*et al*. Treatment of patients with acquired idiopathic generalized anhidrosis. Br J Dermatol
2013; 168: 430–432.2270938110.1111/j.1365-2133.2012.11112.x14

Asahina
M
, 
Sano
K
, 
Fujinuma
Y
, 
Kuwabara
S
. Investigation of antimuscarinic receptor autoantibodies in patients with acquired idiopathic generalized anhidrosis. Intern Med
2013; 52: 2733–2737.2433457610.2169/internalmedicine.52.105015

Fukunaga
A
, 
Hatakeyama
M
, 
Tsujimoto
M

*et al*. Steroid treatment can improve the impaired quality of life of patients with acquired idiopathic generalized anhidrosis. Br J Dermatol
2015; 172: 537–538.2506090310.1111/bjd.1328516
Japan Meteorological Agency
[homepage on the internet]. 2020. Available from URL: https://www.jma.go.jp/jma/indexe.html
17

Benjamini
Y
, 
Hochberg
Y
. Controlling the false discovery rate: a practical and powerful approach to multiple testing. J R Stat Soc Ser B
1995; 57: 289–300.18
R Core Team
. R: A language and environment for statistical computing. 2018. Available from URL: https://www.r‐project.org/
19

Hervé
M
. RVAideMemoire: testing and plotting procedures for biostatistics. R Package. 2020. http://cran.r‐project.org/package=RVAideMemoire
20
Cancer Therapy Evaluation Program, National Cancer Institute: Common Terminology Criteria for Adverse Events (CTCAE) v5.0. 2017. Available from URL: https://ctep.cancer.gov/protocolDevelopment/electronic_applications/ctc.htm
21

Masuda
T
, 
Obayashi
K
, 
Ueda
M

*et al*. Therapeutic effects and prevention of recurrence of acquired idiopathic generalized anhidrosis via i.v. immunoglobulin treatment. J Dermatol
2016; 43: 336–337.2650839210.1111/1346-8138.1318222

Suma
A
, 
Murota
H
, 
Kitaba
S

*et al*. Idiopathic pure sudomotor failure responding to oral antihistamine with sweating activities. Acta Derm Venereol
2014; 94: 723–724.2453511410.2340/00015555-182023

Rice
JB
, 
White
AG
, 
Scarpati
LM

*et al*. Long‐term systemic corticosteroid exposure: a systematic literature review. Clin Ther
2017; 39: 2216–2229.2905550010.1016/j.clinthera.2017.09.01124

Ciuha
U
, 
Kounalakis
S
, 
McDonnell
AC
, 
Mekjavic
IB
. Seasonal variation of temperature regulation: do thermoregulatory responses “spring” forward and “fall” back?
Int J Biometeorol
2020; 64: 1221–1231.3219359510.1007/s00484-020-01898-w25

Taniguchi
Y
, 
Sugenoya
J
, 
Nishimura
N

*et al*. Contribution of central versus sweat gland mechanisms to the seasonal change of sweating function in young sedentary males and females. Int J Biometeorol
2011; 55: 203–212.2053257210.1007/s00484-010-0325-126

Hori
S
. Adaptation to Heat. Jpn J Physiol
1995; 45: 921–946.867657810.2170/jjphysiol.45.921

## Supporting information


**Figure S1**. Receiver–operator curve with age as predictor and recurrence as negative label.
**Figure S2**. Log‐minus‐log plots of Kaplan–Meier estimation for validation of proportional hazard assumption.
**Figure S3**. Rate of response to steroid pulse therapy and humidity.
**Figure S4**. Adapted Kaplan–Meier plot of cure rate.
**Table S1**. Number of patients from each academic medical center
**Table S2**. *P*‐values of all pairs of months, related to Figure 1(b)
**Table S3**. Details of individual course of steroid pulse therapy and its response, related to Table 2
**Table S4**. Adverse effects of steroid pulse therapy
**Table S5**. Comparison of patient characteristics between young and older groups of patients enrolled to time‐to‐event analysis
**Table S6**. Comparison between Minor’s test and self‐assessment of sweating function after steroid pulse therapy
