# Supplementary material for: Prognosis after steroid pulse therapy and seasonal effect in acquired idiopathic generalized anhidrosis
Source: J Dermatol. 2020 Nov 4;48(3):271–8. doi: 10.1111/1346-8138.15666 (PMC7984388; doi:10.1111/1346-8138.15666)
Supplement: Supplementary file 1 — Figure S1. Receiver–operator curve with age as predictor and recurrence as negative label. Figure S2. Log‐minus‐log plots of Kaplan–Meier estimation for validation of proportional hazard assumption. Figure S3. Rate of response to steroid pulse therapy and humidity. Figure S4. Adapted Kaplan–Meier plot of cure rate. Table S1. Number of patients from each academic medical center Table S2. P‐values of all pairs of months, related to Figure 1(b) Table S3. Details of individual course of steroid pulse therapy and its response, related to Table 2 Table S4. Adverse effects of steroid pulse therapy Table S5. Comparison of patient characteristics between young and older groups of patients enrolled to time‐to‐event analysis Table S6. Comparison between Minor’s test and self‐assessment of sweating function after steroid pulse therapy [file JDE-48-271-s001.docx]

**Supporting Information**

**Figure S1**


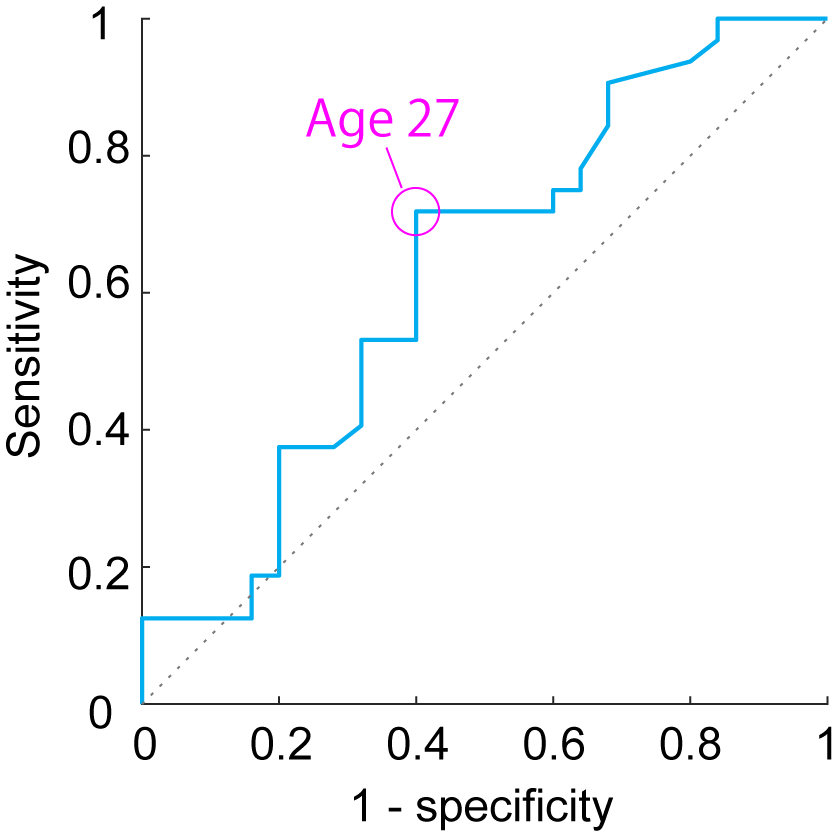


Receiver operating characteristic (ROC) curve with age as predictor and recurrence as (negative) label. To determine optimal threshold value of age, we conducted ROC analysis with age and recurrence data of 57 patients (the same subject as the time-to-event analysis, Table S4). Circle in magenta indicate the optimal value (age of 27).

**Figure S2**


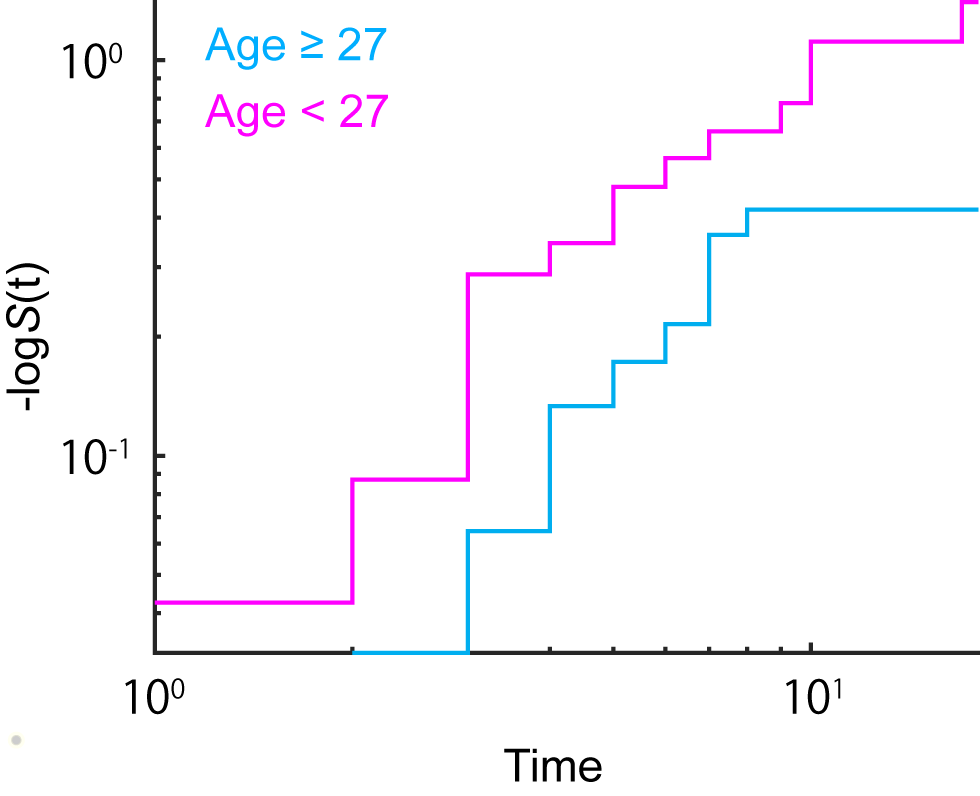


Log-minus-log plots of Kaplan-Meier estimation for validation of proportional hazard assumption. The plots of young and older groups are almost parallel to each other, which indicates the assumption is not violated.

**Figure S3**


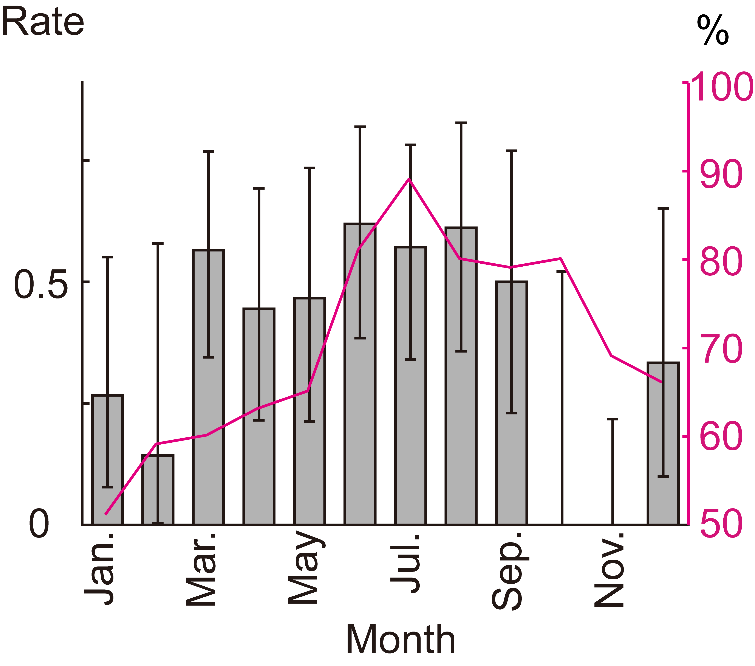


Rate of response to steroid pulse therapy and humidity. The plot in magenta indicates the mean relative humidity of each month in Tokyo, Japan in 2019. Bars are same as Figure 2b indicating the rates of response (error bars indicate 95% CI). Number of courses of pulse therapy: n = 19 (January), 8 (February), 36 (March), 26 (April), 22 (May), 34 (June), 33 (July), 29 (August), 21 (September), 5 (October), 15 (November), and 16 (December).

**Figure S4**

**
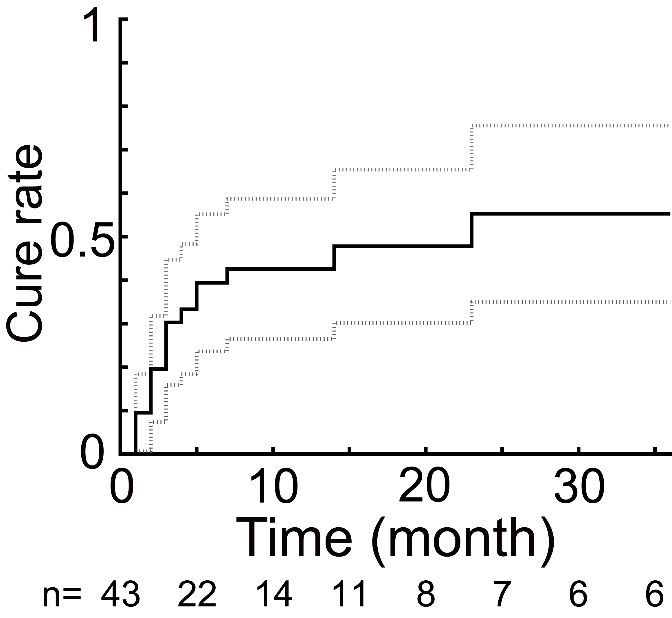
**

Adapted Kaplan–Meier plot of cure rate (dotted plots indicate 95% CI). A time-to-event analysis involving all patients from Tokyo Medical and Dental University was conducted. The traces were started on the first course of steroid pulse therapy and ended at the last course for “cured” patients (18 of 85 [21 %]), or were censored at 11 months before the last consultation day for the rest of patients (25 [29 %] patients were included to the analysis; 42 [49 %] patients were excluded from the analysis because their follow-up periods were less than 11 months). The estimated cure rate reached 55% (95% CI 35-76%) at month 23.

**Table S1. Number of patients from each academic medical center**

|  | All | TMDU^†^ | SMU^‡^ | AMU^§^ |
| --- | --- | --- | --- | --- |
| With steroid pulse therapy | 124 | 85 | 21 | 18 |
| Without steroid pulse therapy | 33 | 26 | 1 | 6 |

Data are the number of patients with acquired idiopathic generalized anhidrosis (AIGA). The response rate of steroid pulse therapy was calculated from the 124 patients from three academic medical centers. †Tokyo Medical and Dental University hospital. ‡Saitama Medical University hospital. §Aichi Medical University hospital.

**Table S2. *P*-values of all pairs of months, related to Figure 2b**

|  | Jan. | Feb. | Mar. | Apr. | May | Jun. | Jul. | Aug. | Sep. | Oct. | Nov. |
| --- | --- | --- | --- | --- | --- | --- | --- | --- | --- | --- | --- |
| Feb. | 1.000 | - | - | - | - | - | - | - | - | - | - |
| Mar. | 0.314 | 0.314 | - | - | - | - | - | - | - | - | - |
| Apr. | 0.786 | 0.669 | 0.786 | - | - | - | - | - | - | - | - |
| May | 0.786 | 0.472 | 0.927 | 1.000 | - | - | - | - | - | - | - |
| Jun. | 0.248 | 0.314 | 0.937 | 0.666 | 0.786 | - | - | - | - | - | - |
| Jul. | 0.314 | 0.314 | 1.000 | 0.786 | 0.927 | 1.000 | - | - | - | - | - |
| Aug. | 0.314 | 0.314 | 1.000 | 0.786 | 0.786 | 1.000 | 1.000 | - | - | - | - |
| Sep. | 0.580 | 0.441 | 0.927 | 1.000 | 1.000 | 0.786 | 0.927 | 0.927 | - | - | - |
| Oct. | 0.786 | 1.000 | 0.240 | 0.336 | 0.326 | 0.240 | 0.240 | 0.240 | 0.318 | - | - |
| Nov. | 0.314 | 0.636 | **0.005** | **0.040** | 0.060 | **0.005** | **0.005** | **0.005** | **0.029** | 1.000 | - |
| Dec. | 1.000 | 0.865 | 0.596 | 0.927 | 0.927 | 0.415 | 0.596 | 0.580 | 0.786 | 0.580 | 0.233 |

Statistical analysis by Fisher’s exact test on monthly rate of response of steroid pulse therapy (Figure **2**b). *P*-values are adjusted by Benjamini-Hochberg method. *P* values of <0.05 are indicated in bold.

**Table S3. Details of individual course of steroid pulse therapy and its response, related to Table 2**

| Number of course | Responder, without recurrence^†^ | | | | Responder, with recurrence^‡^ | Non-responder |
| --- | --- | --- | --- | --- | --- | --- |
|  | once | twice | 3 times^‡^ | ≥4 times^‡^ |  |  |
| 1st | 1.0  (10/10) | 0.38  (6/16) | 0.67  (2/3) | 0  (0/3) | 0.65  (13/20) | 0  (0/26) |
| 2nd | NA | 0.93  (13/14) | 1.0  (3/3) | 0.33  (1/3) | 0.73  (11/15) | 0  (0/14) |
| 3rd | NA | NA | 0.67  (2/3) | 0  (0/3) | 0.50  (6/12) | 0  (0/12) |
| After 4th | NA | NA | NA | 0.80  (4/5) | 0.53  (9/17) | 0  (0/5) |

NA, not applicable. Data are rate of response (n, course of steroid pulse). †Grouped based on the total number of courses required for complete the treatment. ‡When excluding the data from non-responders and responders that completed the treatment with one or two courses, response rates were not significantly different (1st, 58% [15/26]; 2nd, 71% [15/21]; 3rd 44% [8/18]; After 4th, 59% [13/22]; Fisher’s test, *p* = 0.41).

**Table S4. Adverse effects of steroid pulse therapy**

|  | | Grade 1 | Grade 2 | Grade 3 | Grade 4 | Grade 5 | All (%) |
| --- | --- | --- | --- | --- | --- | --- | --- |
| Psychiatric disorders | | | | | | | 13 |
|  | Insomnia | 8 | 0 | 0 | 0 | 0 | 8 (4.3) |
|  | Depression | 0 | 2 | 0 | 0 | 0 | 2 (1.1) |
|  | Psychosis | 0 | 1 | 0 | 0 | 0 | 1 (0.5) |
|  | Agitation | 1 | 0 | 0 | 0 | 0 | 1 (0.5) |
|  | Restlessness | 1 | 0 | 0 | 0 | 0 | 1 (0.5) |
| Gastrointestinal disorders | | | | | | | 10 |
|  | Nausea | 1 | 3 | 0 | 0 | 0 | 4 (2.2) |
|  | Stomach pain | 2 | 0 | 0 | 0 | 0 | 2 (1.1) |
|  | Abdominal pain | 2 | 0 | 0 | 0 | 0 | 2 (1.1) |
|  | Diarrhea | 1 | 0 | 0 | 0 | 0 | 1 (0.5) |
|  | Mucositis oral | 1 | 0 | 0 | 0 | 0 | 1 (0.5) |
| Nervous system disorders | | | | | | | 7 |
|  | Headache | 3 | 0 | 0 | 0 | 0 | 3 (1.6) |
|  | Dizziness | 2 | 0 | 0 | 0 | 0 | 2 (1.1) |
|  | Concentration impairment | 1 | 0 | 0 | 0 | 0 | 1 (0.5) |
|  | Paresthesia | 1 | 0 | 0 | 0 | 0 | 1 (0.5) |
| General disorders and administration site conditions | | | | | | | 5 |
|  | Malaise | 4 | 0 | 0 | 0 | 0 | 4 (2.2) |
|  | Edema limbs | 1 | 0 | 0 | 0 | 0 | 1 (0.5) |
| Skin and subcutaneous tissue disorders | | | | | | | 4 |
|  | Pruritus | 0 | 2 | 0 | 0 | 0 | 2 (1.1) |
|  | Eczema | 1 | 0 | 0 | 0 | 0 | 1 (0.5) |
|  | Pain of skin | 1 | 0 | 0 | 0 | 0 | 1 (0.5) |
| Eye disorders | | | | | | | 3 |
|  | Blurred vision | 2 | 0 | 0 | 0 | 0 | 2 (1.1) |
|  | Eye pain | 1 | 0 | 0 | 0 | 0 | 1 (0.5) |
| Ear and labyrinth disorders | | | | | | | 2 |
|  | Hearing impaired | 2 | 0 | 0 | 0 | 0 | 2 (1.1) |
| Investigations | | | | | | | 1 |
|  | Weight gain^†^ | 0 | 0 | 1^†^ | 0 | 0 | 1 (0.5) |

(Continued to next page)

**Table S4 Adverse effects of steroid pulse therapy (continued from previous page).**

|  | | Grade 1 | Grade 2 | Grade 3 | Grade 4 | Grade 5 | All (%) |
| --- | --- | --- | --- | --- | --- | --- | --- |
| Endocrine disorders | | | | | | | 1 |
|  | Adrenal insufficiency | 0 | 1 | 0 | 0 | 0 | 1 (0.5) |
| Infections and infestations | | | | | | | 1 |
|  | Shingles | 0 | 1 | 0 | 0 | 0 | 1 (0.5) |
| All | | 36 | 10 | 1 | 0 | 0 | 47 |

List of adverse effects seen in the 184 courses of steroid pulse therapy. The adverse effects were categorized and graded according to the Common Terminology Criteria for Adverse Events (v5.0, National Cancer Institute, United States)^20^. Data are presented as n (percentage). †One patient showed >20% weight gain during 2 months of prolonged oral steroids after pulse therapy.

**Table S5. Comparison of patient characteristics between young and older group of patients enrolled to time-to-event analysis**

|  | | All (n = 57) | Age ≺ 27  (n = 24) | Age ≥ 27  (n = 33) | *P*-values^†^ |
| --- | --- | --- | --- | --- | --- |
| Age, years | | 30 (19-40) | 19 (17-20) | 38 (32-43) | NA |
| Gender | |  |  |  | 0.053 |
|  | Male | 43 | 15 | 28 |  |
|  | Female | 14 | 9 | 5 |  |
| Disease score^‡^ | |  |  |  | 0.451 |
|  | 1 | 5 | 2 | 3 |  |
|  | 2 | 8 | 5 | 3 |  |
|  | 3 | 44 | 17 | 27 |  |
| Cholinergic urticaria | |  |  |  | **0.039** |
|  | Yes | 39 | 20 | 19 |  |
|  | No | 18 | 4 | 14 |  |
| Delay between onset and treatment, months | | 0.8 (0.4-1.8) | 0.8 (0.2-2.4) | 0.6 (0.4-1.6) | 0.802 |
| Recurrence | |  |  |  | **0.016** |
|  | Yes | 25 | 15 | 10 |  |
|  | No | 32 | 9 | 23 |  |
| Follow-up time of patient without recurrence, month | | 12 (7-19) | 10 (6-25) | 13 (8-18) | 0.659 |

NA, not applicable. Data are presented as n or median (interquartile range). †For continuous variables, computed by Mann-Whitney *U* test; for categorical variables, computed by Fisher’s exact test. ‡Scored according to body surface area of hypohidrosis or anhidrosis (score of 1, 25%–50%; score of 2, 50%–75%; score of 3, 75%–100%)^1^. *P* values of <0.05 are indicated in bold.

**Table S6. Comparison between Minor’s test and self-assessment of sweating function after steroid pulse therapy**

|  | | Minor's test | |
| --- | --- | --- | --- |
|  |  | Effective | Ineffective |
| Self-assessment | Effective | 20 | 2 |
|  | Ineffective | 4 | 66 |

Data are presented as n (course of steroid pulse therapy). To evaluate the accuracy of the self-assessment at outpatient care, we compared it with the result of Minor’s test performed subsequently at the time of rehospitalization. We collected all the results of Minor’s test performed after steroid pulse therapy from Tokyo Medical and Dental University. Patients were judged as “effective” if the body surface area with perspiration had increased by >25% from the baseline at the time of diagnosis in either approach. Sensitivity, specificity, and accuracy of self-assessment was 83%, 97% and 93%, respectively. There were relatively few effective cases because we usually did not perform Minor’s test for patients who responded well to the therapy and do not want an additional treatment.
